# Phase Equilibria and Structure Formation in the Polylactic-co-Glycolic Acid/Tetraglycol/Water Ternary System

**DOI:** 10.3390/polym15051281

**Published:** 2023-03-03

**Authors:** Polina Yu. Algebraistova, Andrey V. Basko, Anna N. Ilyasova, Tatyana N. Lebedeva, Anton V. Mironov, Konstantin V. Pochivalov, Vladimir K. Popov

**Affiliations:** 1 Institute of Photonic Technologies, Federal Scientific Research Center “Crystallography and Photonics”, Russian Academy of Sciences, Pionerskaya 2, Troitsk, Moscow 108840, Russia; 2 G.A. Krestov Institute of Solution Chemistry, Russian Academy of Sciences, Akademicheskaya 1, Ivanovo 153045, Russia; 3 Institute of Macromolecular Compounds, Russian Academy of Sciences, Bolshoi Prospekt 31, St. Petersburg 199004, Russia

**Keywords:** polylactic-co-glycolic acid (PLGA), tetraglycol (TG), ternary phase diagram, nonsolvent-induced phase separation (NIPS), polymeric scaffolds

## Abstract

This paper concerns a detailed study of the phase separation and structure formation processes that occur in solutions of highly hydrophobic polylactic-co-glycolic acid (PLGA) in highly hydrophilic tetraglycol (TG) upon their contact with aqueous media. In the present work, cloud point methodology, high-speed video recording, differential scanning calorimetry, and both optical and scanning electron microscopy were used to analyze the behavior of PLGA/TG mixtures differing in composition when they are immersed in water (the so-called “harsh” antisolvent) or in a nonsolvent consisting of equal amounts of water and TG (a “soft” antisolvent). The phase diagram of the ternary PLGA/TG/water system was designed and constructed for the first time. The PLGA/TG mixture composition with which the polymer undergoes glass transition at room temperature was determined. Our data enabled us to analyze in detail the structure evolution process taking place in various mixtures upon their immersion in “harsh” and “soft” antisolvent baths and gain an insight into the peculiarities of the structure formation mechanism active in the course of antisolvent-induced phase separation in PLGA/TG/water mixtures. This provides intriguing opportunities for the controlled fabrication of a wide variety of bioresorbable structures—from polyester microparticles, fibers, and membranes to scaffolds for tissue engineering.

## 1. Introduction

The architectonics of the porous structures of polymeric materials produced with the aid of solutions depends on the type of phase decomposition of homogeneous mixtures of a polymer and a low-molecular-weight substance (LM). Phase transformations can be initiated by thermally induced phase separation (TIPS) [1,2,3,4,5,6,7], by adding an antisolvent to these mixtures (nonsolvent-induced phase separation (NIPS) [8,9,10,11,12,13,14]), or through a combination thereof [15,16]. Less often, antisolvent vapor-induced phase inversion [17], as well as the pressure reduction method [18], is used if the required homogeneous mixture is obtained using a supercritical fluid. The resulting porous structures formed from mixtures containing semicrystalline polymers are fixed by crystallization [19,20], while those from mixtures containing amorphous polymers are fixed because of vitrification [21,22].

At the same time, a whole range of various micro- and macroporous structures can be formed in the final product, depending on the ratio between the components of the initial mixture. Such structures can vary from a homogeneous monolith [23,24,25] to a symmetrical cellular topology (which can be obtained mainly by the TIPS method); spongy and asymmetrical structures can also be formed and can contain, in addition, thin dense surface layers or big finger-like pores when use is made of the NIPS method.

We previously used the NIPS approach when developing a new process that we proposed for the antisolvent 3D printing of bioresorbable polymer scaffolds for tissue engineering purposes [26,27]. A solution of hydrophobic polylactic-co-glycolic acid (PLGA) in the nontoxic hydrophilic solvent tetraglycol (TG) was shown to be a promising candidate for the computer-controlled antisolvent 3D printing of biocompatible structures with predefined architectonics.

The high thermodynamic affinity of tetraglycol for water makes it possible to effectively extract TG from polymer solutions, inducing the formation of solid polymeric structures via phase separation. Moreover, a PLGA/TG/water system with certain proportions of the components does not exhibit acute cytotoxicity [28]. This makes it possible to use it for the room-temperature fabrication of various biomedical products capable of immobilizing a wide range of thermolabile bioactive components (drugs, growth factors, etc.) [27].

In our previous work [26], we did not study the thermodynamics of the initial PLGA/TG mixtures, nor the kinetics of the formation of polymeric structures from mixtures differing in polymer content (from 10 to 25 wt.%) undergoing phase separation upon immersion in water at different temperatures (10–40 °C). Using scanning electron microscopy (SEM), we mainly analyzed the surface morphology and cross-sections of structures from 0.1 to 300 μm in characteristic size obtained under various conditions. However, without thermodynamic and kinetic data at one’s disposal for the phase separation process in the systems described above, one cannot develop technological processes for the consistent reproduction of valuable items (membranes, scaffolds, microparticles, etc.) having the required structural and physicochemical properties.

It has been well known that thermodynamic data for such ternary systems can be obtained by analyzing their ternary phase diagrams at a fixed temperature. However, since these diagrams describe the behavior of systems mainly under equilibrium conditions, while structure formation in the case of NIPS is a nonequilibrium process [29], it becomes obvious that the kinetics of mass transfer processes in the systems of interest must also be studied.

There are several methods that have been successfully used to study the kinetics of structure formation in three-component mixtures in the case of NIPS. Chronologically, one of the earliest among them was the turbidimetric technique proposed by C.A. Smolders and co-workers [30,31,32]. Based on the character of the time dependence of the light transmission of the polymer films being formed in the precipitation bath, these authors suggested that there existed two-phase separation mechanisms, one being instantaneous and the other being delayed. One of the significant disadvantages of this approach is that the intensity of light scattered by an emulsion depends, among other things, on the difference in refractive indexes between the fluids forming the emulsion. This does not allow one to compare mixtures of different compositions. Another experimental technique, based on the previous work by R. Matz [33], was successfully used for this purpose in [34,35,36,37,38]. The direct microscopic observation of membrane formation via NIPS was carried out using the polysulfone/N-methylpyrrolidone/water system [34]. The interface between the polymer solution and drops of the antisolvent, which is placed between the slide and the cover glass, was observed and analyzed. In this case, however, the volumes of the polymer solution and the antisolvent were comparable, which is far from the conditions of the NIPS process in actual practice, where the antisolvent bath modulus is usually at least 1:10. Although the effect of the polymer concentration in the initial mixture on the growth rate of the finger-like pores and their length, width, and specific volume was considered in this work, the authors did not speculate on the thermodynamic aspects of the issue.

The main purpose of this work was to provide solutions to the following problems: (1) to construct the phase diagram of the PLGA/TG/water ternary mixture; (2) to describe the thermodynamic behavior of this system; (3) to study the evolution of the forming polymer structure for systems of various compositions upon their contact with hard and soft antisolvents; (4) to develop a clear physical understanding of the structure formation mechanism during NIPS.

## 2. Materials and Methods

### 2.1. Materials

**Figure 1:**
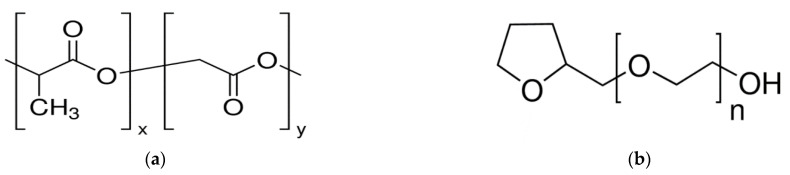
Structural formulas of (**a**) PLGA (x = 0.75, y = 0.25) and (**b**) (*n* = 3).

Biocompatible and bioresorbable polylactic-co-glycolic acid (PLGA) Grade Purasorb PLGA 7507 (Corbion Purac, Amsterdam, The Netherlands) with an inherent viscosity midpoint of 0.2 dL/g and a lactic-to-glycolic-acid monomer ratio of 75:25 (Figure 1a) was used as a raw polymeric material for forming polyester structures. Tetrahydrofurfuryl alcohol polyethylene glycol ether (TG) Grade BioXtra (Sigma Aldrich, St. Louis, MO, USA) was used as a nontoxic organic solvent for this polymer (Figure 1b).

### 2.2. Investigation Methods

#### 2.2.1. Constructing the Phase Diagram of the Ternary PLGA/TG/Water Mixture

To construct the phase diagram of the three-component mixture, we used the well-known cloud point method [39,40,41]. The experimental methodology was as follows. The PLGA/TG mixture of the required composition and known mass, preliminarily prepared in a weighing bottle at 80 °C and homogenized in an ultrasonic bath (Model RK 102 H Sonorex Super; Bandelin Electronic, Berlin, Germany), was cooled to room temperature and kept at this temperature for 2 h. Thereafter, water at the same temperature was injected with a syringe, drop by drop, into the bottle, each drop causing local opalescence in the mixture, which vanished some time later. Water injection was continued until stable opalescence developed throughout the volume of the bottle. The amount of each component of the mixture at that moment was determined by weighing with an analytical balance.

#### 2.2.2. Differential Scanning Calorimetry (DSC) of the Binary PLGA/TG Mixtures

The DSC thermograms of the PLGA/TG mixtures differing in composition were obtained with a Phoenix 204F1 (NETZSCH, Waldkraiburg, Germany) differential scanning calorimeter (temperature scanning speed 10 °C/min, mass of the samples sealed into crucibles 3–7 mg, standard calibration). Experiments were performed as follows. Samples of various PLGA/TG mixtures, as well as of pure PLGA and TG, sealed into crucibles, were cooled to −70 °C, heated to 120 °C, cooled once more to −70 °C, and heated again to 120 °C, with the crucibles with samples being kept for 5 min at each of the temperatures indicated above.

#### 2.2.3. Studying the Evolution of the Structures Formed from PLGA/TG Mixtures upon Their Contact with a Nonsolvent

The experimental methodology was as follows. A drop (about 40 μL volume) of a preliminarily prepared homogeneous PLGA/TG mixture of the required composition under study was placed on a slide between side fences formed thereon by two strips of a polyimide film, around 50 μm in thickness, arranged parallel to each other. The distance between the strips was around 30 mm, and their length was around 90 mm. The glass slide with the drop of the mixture between the side fences at its center was covered with cover glass. This arrangement was clamped with spring clips and placed on the stage of an optical microscope (Model Micromed C-11, NP Ltd., St. Petersburg, Russia). Thereafter, distilled water mixed with Grade E133 dye (Dynamic Products Ltd., Ahmedabad, India) was injected with an insulin syringe between the slides so that it filled the entire fenced-in space. Such a “cell” design allowed us to achieve a bath modulus of ~1:50. The events that were taking place during the course of the contact between the mixture drop and water were recorded with a Model Poco X3 Pro (Beijing, China) digital camera.

#### 2.2.4. Methodology for Obtaining Fibers

Polymer fiber samples were formed by extruding 10, 20, and 30 wt.% PLGA/TG solutions through a 280 μm dia. nozzle into a precipitation bath of either distilled water or a 25, 50, or 75 wt.% aqueous TG solution using a specially designed laboratory 3D printer [42]. Upon the fixing of the PLGA structure, the samples were placed in distilled water for 24 h to extract TG residues from them, then rinsed twice with distilled water, and dried in air at 25 °C.

#### 2.2.5. Studying the Morphology of the Polymer Structures and Fibers Being Formed

The microstructures of the samples (cross-sections and surfaces) were studied with scanning electron microscopes (Models Phenom ProX (Phenom, Utrecht, The Netherlands) and Quattro S (Thermo Fisher Scientific, Černovice, Czech Republic)). In the former case, dried fiber samples free from additional metal coatings were held fast to the microscope stage with a conductive carbon ribbon. The study was performed at a cathode voltage of 15 kV. In the latter case, to study the surfaces of fibers at a high resolution, the fibers were coated with gold using a Model Quorum Q150 plus sputter coater (Czech Republic), and a cathode voltage of 5 kV was used.

## 3. Results and Discussion

### 3.1. DSC Studies of PLGA/TG Mixtures

**Figure 2:**
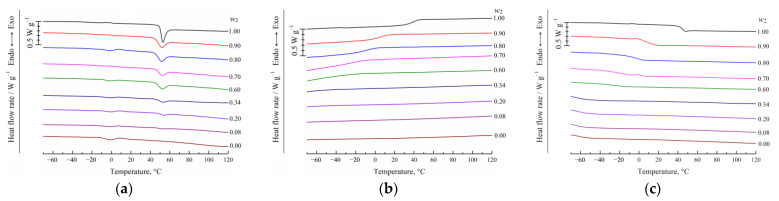
Thermograms of PLGA, TG, and their mixtures (PLGA mass fraction indicated on the right of the curves) obtained in the course of (**a**) the first heating session, (**b**) first cooling session, and (**c**) second heating session.

PLGA is an amorphous polyester, so the DSC technique allows one to evaluate the effect of the TG dissolved in the polymer on its glass transition temperature *T_g_*. The thermograms for pure PLGA, pure TG, and the PLGA/TG mixtures of various compositions, obtained in the course of their heating–cooling–heating sessions, are shown in Figure 2.

It can be seen from this figure that the thermograms for the samples of pure PLGA and PLGA/TG mixtures in the composition range 0.20 ≤ *w*_2_ ≤ 1, obtained during the first heating session (Figure 2a), feature an endothermal peak superimposed on the step in the heat capacity variation curve of the polymer, which characterizes its glass transition (*T_g_*), this reflecting, as follows from the works by Lee and co-workers [43] and Reich [44], the processes of the relaxation of the structure of the amorphous polymer. That the glass transition temperature *T_g_* was independent of the parent mixture composition suggested that the long dwell time of the mixture in a sealed crucible at room temperature (around 24 h) did not result in the formation of a homogeneous solution of the low-molecular-weight component in the copolymer. The small endothermal peak at ~0 °C that is evident in all the thermograms reflects the melting of the water sorbed by the sample (the copolymer or TG).

At the same time, it has been well known [7] that the initial heating, followed by soaking at an elevated temperature, provides homogeneous mixtures, and the subsequent cooling in the case where they undergo phase decomposition ensures a manifold increase in the contact surface between the components, hence shortening the duration of the diffusion processes taking place in the course of the second heating session.

It is exactly for this reason that use has traditionally been made, when discussing DSC data, of endotherms for mixtures obtained in the course of the second heating session and exotherms for those resulting from the first cooling.

One can see from Figure 2c that the endothermal peak reflecting the thermal effect due to the relaxation of the amorphous structure of the polymer in the thermograms for the samples obtained during the second heating session is practically degenerate, while the temperature corresponding to the midpoint of the glass transition in the thermograms, which corresponds to the temperature *T_g_*, decreases as the TG content of the mixture is increased from 0.11 to 0.29 wt.%. As the TG content of the mixture was further increased, the instrument no longer recorded the glass transition.

When cooling mixtures with compositions within this range (Figure 2b), the respective thermograms also feature an inflection, reflecting the sharp change in heat capacity due to the vitrification of the polymer, and the midpoint temperature also decreases with increasing TG concentration in the mixture.

**Figure 3:**
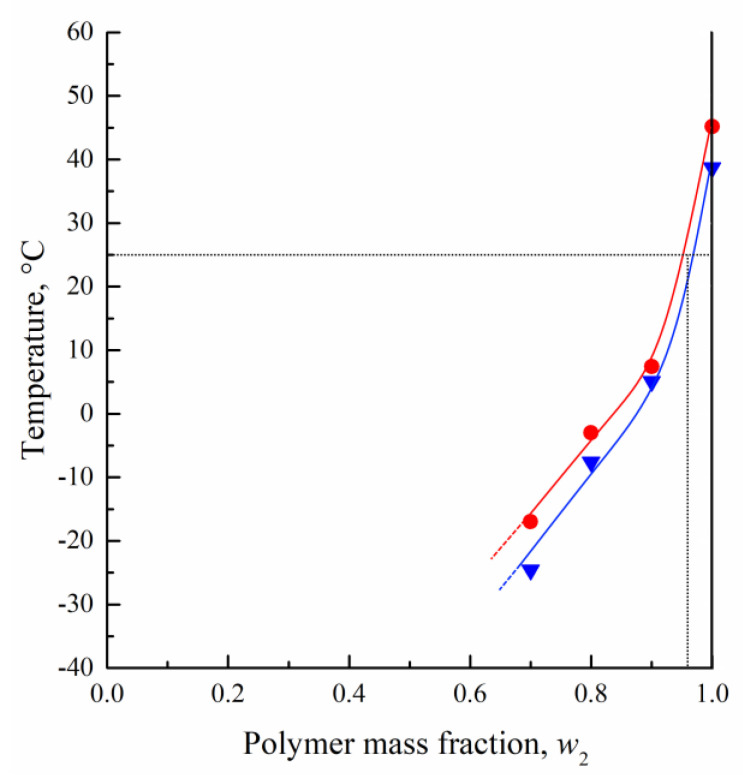
Glass transition temperature *T_g_* of PLGA/TG mixture samples as a function of their PLGA content, determined by the DSC method during the first cooling (blue triangles) and second heating (red circles) sessions.

Figure 3 illustrates the lowering of the *T_g_* of PLGA as a result of the dissolution of TG therein. It can be seen that, as expected, the *T_g_* values for the samples obtained upon cooling are systematically below those for their counterparts resulting from repeated heating. The data displayed in this figure allow the determination, in a first approximation, of the conditions conducive to the fixing, as a result of vitrification, of the structures formed via NIPS of PLGA/TG mixtures.

### 3.2. Phase Diagram of the Ternary PLGA/TG/Water Mixture

To evaluate the thermodynamic affinity between the components of polymer-containing systems, use is made of various approaches, such as those requiring the estimation of the second virial coefficient [45], the osmotic pressure of the solution [46], etc. However, the approaches by Hildebrand [47] and Hansen [48], based on comparing the solubility parameters of the respective substances, have found the widest applications, being customarily assumed that the smaller the difference between the solubility parameters of two substances, the higher the level of their thermodynamic affinity. Given as the data on the solubility parameters of substances published in the middle of the XX century cannot be used to estimate such parameters of newly synthesized compounds, group contribution techniques [49,50,51] have been proposed to calculate them. To evaluate the thermodynamic affinity between the components of our system, we used the method and data reported by Stefanis and Panayioton [51]. The solubility parameters of PLGA were taken to be the sums of the products of the respective solubility parameters of lactic acid and glycolic acid by their molar proportions in the copolymer.

The results of the relevant calculations are listed in Table 1.

It should be noted that though the difference in the solubility parameters between TG and water is rather great, these components are miscible in any proportion, as are many other pairs capable of forming hydrogen bonds [5]. As follows from the data in Table 1, good thermodynamic affinity between components occurs in the PLGA/TG and TG/water pairs, whereas the components of the PLGA/water pair are practically incompatible: i.e., the ternary PLGA/TG/water system under study is classified as an “amorphous polymer–solvent–nonsolvent” type.

**Figure 4:**
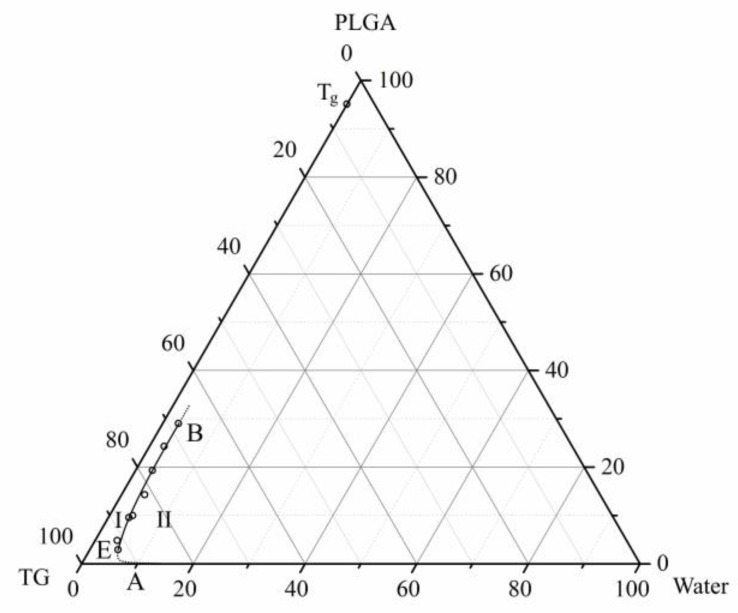
Ternary phase diagram for the PLGA/TG/water mixture at 25 °C. The point *T_g_* plotted on the PLGA–TG axis corresponds to the mixture composition with which the polymer undergoes glass transition at 25 °C.

Figure 4 presents the phase diagram of the ternary PLGA/TG/water mixture at 25 °C, constructed using the methodology described in Section 2.2.1 and Section 2.2.2. Plotted on the same diagram is the point *T_g_*, corresponding to the composition of the binary PLGA/TG mixture in which the polymer undergoes vitrification at 25 °C, found by intersecting the isotherm at this temperature (the dashed line) with the glass transition line in Figure 3.

The phase diagram features a boundary line—the liquid–liquid equilibrium binodal AEB that delimits two regions: I—the single-phase region, wherein there exist homogeneous three-component mixtures; II—the two-phase region, wherein there exists a solution of the polymer dissolved in a two-component mixture of fluids and a solution of such a mixture dissolved in the polymer. The compositions of the phases existing in region II are specified by branches AE and EB of the binodal.

Topological analysis of the above phase diagram allows the representation of the evolution of the structure of PLGA/TG mixtures differing in composition, which occurs upon their immersion in water at room temperature, as follows. When the parent binary PLGA/TG mixture is immersed in the nonsolvent, there takes place mutual diffusion of water into this mixture and of TG out of it into water because of the high thermodynamic affinity between the diffusing components (see Table 1). As a result, there forms a homogeneous three-component PLGA/TG/water mixture.

As these mass-exchange processes continue, the composition of the homogeneous three-component mixture first reaches values corresponding to a point on the liquid–liquid equilibrium binodal AEB (Figure 4), and then its microscopic phase decomposition occurs to form an emulsion consisting of two phases, a polymer-rich phase and a polymer-poor phase, with the compositions of these phases being specified, depending on the variation in the thermodynamic affinity (TG-to-water proportion) between the polymer and the two-component fluid, by branches AE and EB of the binodal.

The morphology of this emulsion depends on the proportion between the components in the parent PLGA/TG mixture. If the amount of the polymer in this mixture is below that corresponding to the critical point E on the binodal, there forms an emulsion of drops of the polymer-rich phase in the polymer-poor one; otherwise, there forms, as a result of phase inversion, an emulsion of drops of the polymer-poor phase in the polymer-rich phase. Where the composition of the parent mixture corresponds to a region in the vicinity of point E, the emulsion formed will have a bicontinuous morphology.

When the composition of the polymer-rich phase becomes, as a result of the above-mentioned mass-exchange and phase decomposition processes, such that the polymer becomes glassy in the presence of the TG and water it contains, the morphology of the emulsion formed is fixed.

It can be seen from the diagram in Figure 4 that in the extreme case corresponding to the absence of water in the mixture of the components, PLGA turns glassy at room temperature when its amount in this mixture is no less than 95 wt.%.

Thus, the practical importance of the diagram obtained under almost equilibrium conditions is that it predicts the region of mixture compositions wherein liquid–liquid phase separation occurs and makes it possible to formulate the above-mentioned general ideas about the events taking place when a polymer/solvent mixture comes into contact with a precipitator. It is, however, obvious that to elucidate the architectural specifics of the porous structures formed, which depend on the relationship between the mass-exchange processes mentioned above, it is necessary to perform additional optical experiments.

### 3.3. Structure Formation in PLGA/TG Mixtures Induced by Nonsolvents Differing in Activity

To study this issue, we used either pure water or TG mixed with water in various proportions as the nonsolvent, with water being considered a “harsh” nonsolvent and the TG/water mixture being regarded as a “soft” nonsolvent.

The advantage of the above-described methodology of studying the structure formation process taking place when polymer/solvent mixtures come into contact with a nonsolvent over the well-known [33,36] methodologies of the direct observation of the NIPS process is the fairly high precipitation bath modulus (of the order of 1:50). This allows the solvent concentration in the nonsolvent bath to be considered negligibly low, which is extremely important, because the driving force of the diffusion of the solvent into the nonsolvent bath and that of the nonsolvent into the sample mixture immersed in the bath weakens with increasing solvent concentration therein.

The structure formation process under study was observed under the optical microscope for as long as was necessary for the front of the structure being formed to advance in the radial direction to a depth of no less than 150 μm from the boundary of the sample. This distance was chosen because the characteristic diameter of the fibers previously used by us [26,52] to form scaffolds by the antisolvent 3D-printing method was of the order of 200–300 μm.

**Figure 5:**
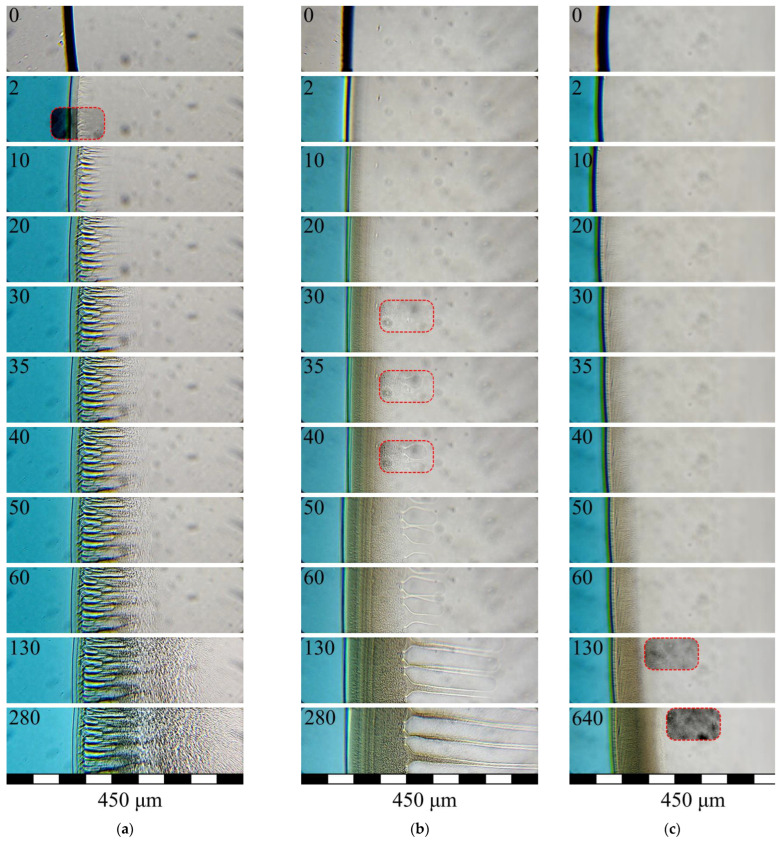
Photographs illustrating the evolution of the structure of PLGA/TG mixtures containing (**a**) 10, (**b**), 20, and (**c**) 30 wt.% polymer upon their contact with a precipitator (stained water). Indicated in the top left corner of each photograph is the time lapsed after the sample mixture came into contact with the precipitator. In the regions delimited by the red dashed lines, the contrast of the image is increased and the brightness is decreased for better visual perception.

**Figure 6:**
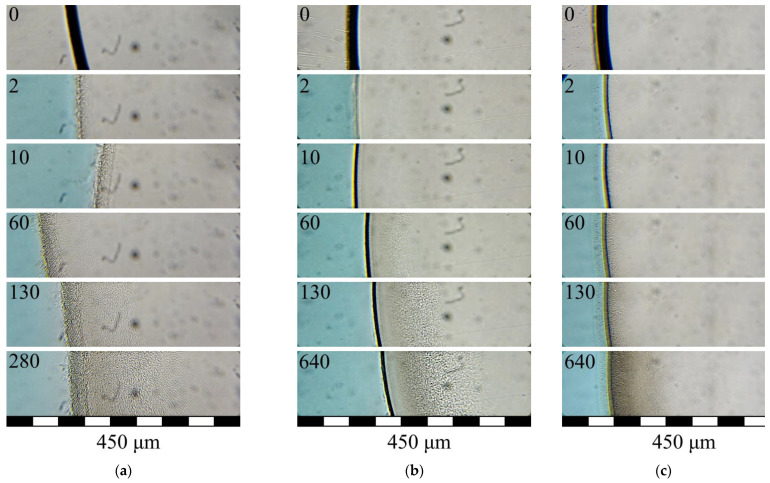
Photographs illustrating the evolution of the structure of PLGA/TG mixtures containing (**a**) 10, (**b**) 20, and (**c**) 30 wt.% polymer upon their contact with the nonsolvent (stained water mixed with TG in the 1:1 ratio). Indicated in the top left corner of each photograph is the time elapsed after the sample mixture came into contact with the nonsolvent.

The photographs in Figure 5 and Figure 6 demonstrate the evolution of a polymer structure in samples of PLGA/TG mixtures of various compositions upon their being immersed in water (Figure 5) and in a mixed nonsolvent (Figure 6).

One can see from Figure 5a that practically immediately at the moment water comes into contact with the edge of the sample (a drop of mixture containing 10 wt.% PLGA) there forms a thin surface layer (the black line). The observed extension (thickness) of this layer is associated with the existence of a meniscus formed in the sample as a result of the compression of the fluid drop between two slides. After less than 2 s, finger-like pores are observed to emerge beneath this layer and continue to grow up to the 35th second of observation. The moment these pores appear, the surrounding matrix starts to get cloudy, reflecting the microscopic liquid–liquid phase separation of the three-component PLGA/TG/water mixture, the front of the clouding process being observed to progress toward the center of the sample throughout the observation time (even after the finger-like-pore growth came to an end). In all cases (Figure 5a–c), the intensity of clouding decreased over time in the direction from the surface toward the center of the sample.

Observed in the sample mixture containing 20 wt.% polymer (Figure 5b) was the clouding of the surface of the sample at the moment it came into contact with water, also reflecting the microscopic liquid–liquid phase separation of the mixture, and the subsequent progress of the clouding front in the direction normal to the sample surface. Within approximately 30 s of the start of observation, there appeared rather big finger-like drops at a distance of approximately 110 μm from the interface, which continued to grow until the termination of the experiment, with the progress of the clouding front continuing in the regions between the growing drops.

It can be seen from Figure 5c that observed throughout the experiment with the PLGA/TG mixture containing 30 wt.% polymer upon contact with water was only the progress of the clouding front toward the center of the sample.

The events observed to take place when immersing PLGA/TG mixtures differing in composition in a mixed nonsolvent consisting of water and TG were identical to those illustrated in Figure 5c, the only difference being that the sample mixture layer bordering the nonsolvent proved hazier (diffuse).

What is more, observed with mixtures containing 10 and 20 wt.% PLGA was a substantial distortion of the shape of the sample mixture drop. This was obviously due to the fact that its surface layer was not enriched with the polymer quickly enough because of the poor extraction power of the nonsolvent and proved, for this reason, less viscous by comparison with the layer formed upon the immersion of the sample in water. The distortion of the shape of the sample made it necessary to shift the position of the optical cell with the sample on the microscope stage in order to observe the structure formation process.

**Figure 7:**
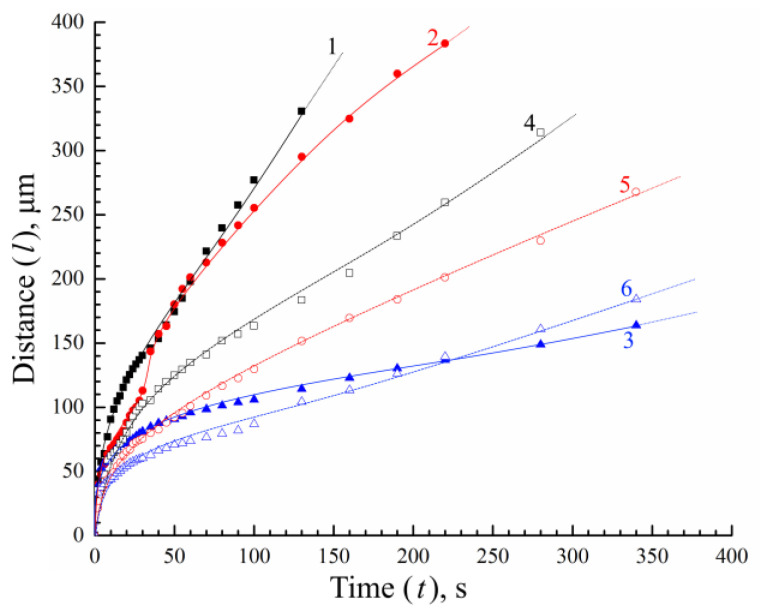
Time dependence of the depth of the structure formation front (solid dots: nonsolvent–pure water; hollow dots: nonsolvent–TG mixed with water in the 1:1 ratio). Black dots refer to the parent mixture containing 10 wt.% PLGA; red dots—20 wt.% PLGA; blue dots—30 wt.% PLGA.

Figure 7 illustrates the time dependence of the depth of the structure formation front (the distance from the sample–nonsolvent contact surface to the clouding front progressing deep into the sample) for all of the PLGA/TG mixtures studied upon their immersion in water and a mixed nonsolvent.

It can be seen that, in most cases (curves 1, 3–6), the function *l* = *f* (*t*) increases monotonously and has no substantial flexures. This means that the rates of progress of the structure formation front monotonously decrease over time, despite the fact that within 35 s after the start of the observation of the mixture containing 10 wt.% PLGA (curve 1), the “fingers” stop growing and the structure formation process continues only by the microscopic fluid phase decomposition mechanism (Figure 5a).

The bend occurring in the kinetic curve after 30 s of observation for the mixture with 20 wt.% PLGA in contact with water (curve 2) reflects an abrupt change in the rate of progress of the structure formation front at the moment the finger-like pores started to grow.

The analysis of the curves presented in Figure 7 allows the following conclusions to be drawn.

**Figure 8:**
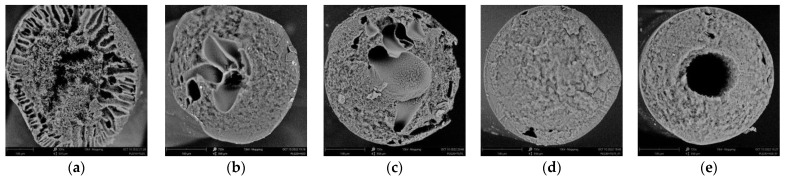
Internal structure of fibers formed from PLGA/TG mixtures via immersion into water and a TG/water mixture. PLGA concentration: (**a**) 10 wt.%, (**b**,**c**) 20 wt.%, (**d**,**e**) 30 wt.%. Nonsolvent: (**a**,**c**,**e**) water and (**b**,**d**) 75% aqueous TG solution.

- When the amount of PLGA in the structure-forming mixture is increased, as well as when water is replaced with a mixed nonsolvent (i.e., the activity of the nonsolvent is reduced), the rate of structure formation tends to decrease, with the rate of progress of the structure formation front being more susceptible to the polymer concentration than to the precipitators’ virtue.
- The lowering of the rate of progress of the structure formation front in the case of the application of the mixed nonsolvent is accompanied by the slowing down of the tempo of the growth of the viscosity of the layer in contact with the fluid. A decrease in the rate of structure formation and a change in the volumetric distribution of the resulting phases with an increase in polymer concentration is associated with an increase in the viscosity of the mixture under study, which varies from ~1.0 Pa·s to more than 50 Pa·s. [26]. This gives us reason to believe that use can be made, in antisolvent 3D printing, of more concentrated structure-forming solutions, compared to those previously used [26] by us. This, in particular, will allow for improving the strength of the finished products and reducing the porosity of their components (fibers). That this supposition is valid is supported by the SEM photographs of the surface and the cleavage of a fiber obtained from a PLGA/TG mixture containing 30 wt.% PLGA (Figure 8) in a bath of a mixed nonsolvent.
- The intersection of curves 4 and 5 at the 50th second, as well as that of curves 5 and 6 at the 220th second, indicates that, prior to the intersection, the rate of progress of the structure formation front is governed largely by the driving force of the mass-exchange process, which weakens when water is replaced with a mixed nonsolvent, while after the intersection, the barrier properties of the surface layer are more expressed in the case where the polymer solution is immersed in water (Figure 5).
- The results of our “optical” experiments using nonsolvents stained with Grade E133 dye show that it takes around 60 s for the concentrations of the LM components in the nonsolvent bath and in the pores of the structure being formed to equalize (although the structure formation front proper progresses deep into the sample rather rapidly—at an average speed of the order of a few microns per second). This means that though PLGA fibers are formed in the antisolvent 3D-printing process quite quickly, they should be kept in the nonsolvent bath long enough to free them completely from TG.

### 3.4. Structure Formation in Amorphous Polymer/Solvent/Nonsolvent Mixtures via NIPS

Considering the results obtained by means of optical microscopy and the topology of the phase diagram (Figure 4), the structure formation mechanism active in PLGA/TG mixtures upon their immersion in the nonsolvent may be formulated as follows.

When a PLGA/TG mixture comes into contact with water, there occur two mass-exchange processes, namely, the diffusion of the solvent out of the sample and the diffusion of the nonsolvent into the sample, and the transformation (initiated by these two processes) of the state of the polymer from viscous-flow to glassy and the resultant fixing of the porous structure being formed.

It is obvious that the rates of these processes depend on the polymer concentration in the structure-forming solution, the activity of the nonsolvent, the coordinate in the cross-section of the sample, and the duration of its contact with the nonsolvent. With the composition of the parent structure-forming solution being fixed, variations in these rates as a function of the occurrence coordinate and time govern the relationship between the components of the ternary mixture being formed across the sample at every instant of time and, in the final analysis, the morphology of the products obtained via NIPS.

Consider the formation specifics of each of the three types of structures fixed by us (Figure 5 and Figure 6). Taking into account the fact that, in the system under discussion, the solvent and nonsolvent have high thermodynamic affinity for each other, while the polymer and nonsolvent are practically incompatible, when a mixture of this system, no matter what its composition, comes into contact with water, the rate of solvent diffusion out of the sample surpasses that of nonsolvent diffusion into the sample.

By virtue of this fact, a thin surface layer of the structure-forming solution gets very rapidly enriched with the polymer and, to an insignificant degree, water. Therefore, the composition of the ternary PLGA/TG/water system being formed in this layer fist reaches the condition corresponding to the top branch (EB) of the liquid–liquid equilibrium binodal (Figure 4) and then enters region II delimited by this curve.

The microscopic liquid–liquid phase separation of the polymer-rich mixture in the surface layer, which takes place in this region, leads to the formation of an emulsion of drops of the polymer-poor phase, whose composition is specified by a point on the bottom branch (AE) of the binodal, in the polymer-rich phase having a composition defined by a point on the top branch (EB) of the binodal. As the solvent–nonsolvent mass-exchange processes continue, the compositions of the existing phases shift along both branches of the binodal. The polymer-poor phase increases in volume at that and tends toward a polymer-free composition, while the polymer-rich one shrinks and reaches a composition with which the polymer becomes glassy, and the porous structure formed gets fixed. As a result, on the sample surface, there forms a thin structure layer containing both closed and open (through) pores. As regards the parent structure-forming solution, irrespective of its composition, this structure layer starts to hinder the ongoing solvent–nonsolvent mass transfer processes. It is quite understandable that the mass transfer of water in the through-pores formed, where it directly contacts the water/TG mixture contained therein, occurs at a substantially higher rate than its diffusion through the polymer matrix containing dissolved TG. As a result of such a nonuniform mass transfer of water in the case of the mixture containing 10 wt.% PLGA, the phase separation process in the ternary mixture first occurs adjacent to the mouths of through-pores to form relatively big drops (nuclei of finger-like pores) of the polymer-poor phase (a TG/water mixture in the extreme case) and then in the mixture behind those areas of the surface structure layer, which are free from through-pores.

Because of the water’s high transfer rate, the mixture of the two liquids contained in the nuclei of finger-like pores becomes oversaturated with water, thus disturbing the thermodynamic equilibrium between the coexisting phases. The dissolution of the surplus water formed in the surrounding polymer matrix containing TG initiates phase separation therein, which is accompanied by the following two processes: the growth of the initially formed finger-like drops toward the center of the sample, thanks to the concurrent addition to their volume of the polymer-poor phase and water coming from the orifices of through-pores (Figure 5a, 2 s), and the formation of an emulsion of small drops of the polymer-poor phase in the polymer-rich one between the “fingers” in the matrix (Figure 5a, 10 s).

At the same time, the mass transfer process taking place beneath the solid, pore-free, surface structure layer is only accompanied by the formation of the above-mentioned emulsion. This event is seen under the microscope as the clouding of the region adjacent to the surface layer. At that, phase decomposition in this layer itself continues, as a result of which its total porosity increases, closed pores interconnect, and, correspondingly, new through-pores form. This means that the rate of water supply through these regions increases and becomes comparable to that of water supply into the “fingers”. The moment these rates equalize, the “fingers” stop growing, and liquid–liquid phase separation at all points proceeds by one and the same mechanism as that observed to occur in the regions between the “fingers”, i.e., by the microscopic liquid–liquid phase separation.

The constant inflow of water into the sample and the outflow of the solvent led to the uninterrupted microscopic phase decomposition in each ternary liquid layer following the interface. Such a continuity of the phase separation process was manifested by the progress of the clouding front toward the center of the sample at an increasing rate relative to the growth rate of the “fingers” until the moment they stopped growing (Figure 5a, 35 s), with the compositions of the coexisting phases in the layers already traversed by the structure formation front varying in accordance with the positions of their characteristic points on the branches of the liquid–liquid equilibrium binodal. In our optical experiments, this was revealed by the fact that the light-scattering intensity was at its maximum near the surface structure layer and decreased to its minimum at the boundary of the structure formation front. Such a gradient was caused by the following two factors: the increasing difference in refractive indexes between the two phases making up the emulsion, whose compositions constantly change over time, and both coalescence and Ostwald ripening in the initially formed emulsion. When the composition of the polymer-rich phase becomes such that the polymer undergoes glass transition at 25 °C, the structure formed in the current section of the structure-forming mixture gets fixed. It is therefore obvious that in the wake of the clouding front, there follows a front at whose boundary the porous structure being formed becomes fixed.

What has been set forth above means that the architecture of the porous structure (the shape and size of pores, their connectivity, spatial arrangement, etc.) is being formed throughout the period starting with the moment the structure-forming solution comes into contact with the nonsolvent and ending with the moment the polymer in the system turns glassy.

At the final formation stage of the structure of the polymer sample, there takes place the replacement of its TG residues by practically pure water. However, when analyzing the results of our optical experiments, we found that this process could take up to 60 min.

Referring now to the mixture containing 20 wt.% PLGA, the structure formation scenario changes only slightly. By analogy with the above-considered case, when this mixture comes into contact with water, the solvent is extracted from the sample into the bath containing the nonsolvent, while the latter diffuses into the sample, and, as a result, the contact layer also becomes enriched with both the polymer and the nonsolvent. However, since the initial polymer concentration was increased, microscopic phase separation within this layer results in the formation of closed pores only.

This means that, initially, the mass transfer of water through this layer to all points in the following layers proceeds uniformly (at equal rates). Since it was exactly the difference in the water supply rate between different points in the structure-forming mixture layers following the surface layer that was the cause of the formation of different structures (finger-like pores and thin emulsion), in the conditions under discussion, there forms but a single type of structure. In other words, all of the water that has diffused through the initial structure layer is consumed to initiate the microscopic liquid–liquid phase separation of the mixtures in the following layers, resulting in the formation of a spongy (bicontinuous) structure similar to the above-discussed thin emulsion between the finger-like pores. As the structure formation front progresses further from the surface structure layer, the compositions of the phases coexisting in the current layers vary in accordance with the scenario described above. The events considered are manifested by the clouding of the current layer (Figure 5b, 2 s) and the progress of the clouding front toward the center of the sample (Figure 5b, 10–30 s). Concurrent with the progress of the structure formation front, in the surface structure layer, the phase separation of the structure-forming mixture continues, accompanied by the formation (emergence) of through-channels in this layer. This facilitates the supply of water to the layers at a distance of the order of 110 μm from the surface. As a result, in the orifices of these channels, there form relatively big drops of the polymer-poor phase, i.e., the nuclei of finger-like pores, and the subsequent structure formation progresses by the above-described mechanism. 

The third type of structure, distinguished by the absence of finger-like pores, was found to form in the mixture containing 30 wt.% PLGA and also in all the mixtures under investigation prepared by the immersion of the mixtures into a mixed nonsolvent made up of TG and water. Despite the similarity in appearance seen under the optical microscope between the structures being formed, their formation mechanisms differ in principle.

In the former case, the increase in the PLGA content of the structure-forming mixture results in no through-channels developing, throughout the structure formation period, in the structure formed in the surface mixture layer because of its enrichment with the polymer via the extraction of the solvent. As a consequence, water comes through this layer to the internal layers of the mixture uniformly at one and the same rate, lower than in the preceding cases (see Figure 5c, 2–640 s), inducing therein uninterrupted microscopic phase separation. In other words, the structure of the sample under discussion is formed by the microscopic phase separation mechanism. This statement is supported by the fact that the permeability of membranes produced by the NIPS method from mixtures of this kind containing over 25 wt.% polymer tends to zero [53,54], which points to the absence of through-pores on their surface.

In the latter case, because of the lower ability of the nonsolvent to extract the solvent, the surface layer of the PLGA/TG mixture samples gets enriched with the polymer to a lower degree. As a result, the structure being formed therein via microscopic phase separation develops a great number of through-channels. Such an open-pore structure of this layer ensures a uniform transfer of water to the subsequent layers. This initiates the microscopic phase separation of the mixture in the layer next to the surface layer (Figure 6a–c, 2 s), and then, as the mass transfer process further continues, the microscopic phase separation front progresses toward the center of the sample. As a result, there originate spongy (bicontinuous) structures free from big finger-like pores.

It should be noted that despite the absence of low-porosity surface layers in the samples under discussion, the rate of progress of the structure formation front in them is lower than that in the case where the samples are immersed in water. This is obviously due exclusively to the weakening of the thermodynamic force driving the mass transfer processes and not to the high levels of the barrier properties of the surface structure layer.

From the foregoing results, it follows that structure formation in items obtained by the NIPS method from mixtures of amorphous polymers and solvents is rooted in the microscopic phase separation process, followed by the fixing of the structures formed, thanks to the glass transition of the polymer. The formation of a porous structure by this method is a nonequilibrium process, and the architecture of the structure depends on the barrier properties of its surface layer that originates when the structure-forming mixture comes into contact with the nonsolvent, with these properties being, in turn, dependent on the morphology of this layer. The morphology of the surface structure layer is governed by the thermodynamic affinity between the nonsolvent used and the solvent in the structure-forming mixture, the polymer content of the mixture, and the diffusion coefficients of the liquids involved in the mass-exchange processes. 

In summary, it should be noted that the notions published in the literature about the mechanism underlying the formation of finger-like pores and the termination of their growth are contradictory. To wit, the authors of [55,56,57,58] attribute the origination and growth of “fingers” to a rapid solvent–nonsolvent exchange, whereas essentially the opposite conclusion is made by those of [12,37,59,60,61]. These authors explain the growth of finger-like pores by the lengthening (owing to low rates of mass-exchange processes) of the residence time of the ternary mixtures in the liquid–liquid phase decomposition region, hence the lengthening of the time during which fine drops of the polymer-poor phase can possibly coalesce. The termination of the growth of “fingers” is, as a rule, attributed to the fixing of the polymer [12,62,63,64].

However, these notions are not quite in agreement with our experimental data presented in Figure 5 and Figure 6.

1. In Figure 5a,b, one can see that the growth front of the finger-like pores outpaces the clouding front that reflexes the microscopic phase separation of the ternary mixture. This means that the formation of the finger-like pores cannot stem from the coalescence of the drops of the polymer-poor phase already in existence.
2. It is evident from Figure 5a,b that with the rates of progress of the structure formation front being nearly the same and relatively high, different structures, in principle, can form.
3. In Figure 5a, it is seen that the structure formation process in the ternary mixture in hand continues even after the “fingers” have stopped growing. This means that the composition of the ternary mixture in the layer following that where the “fingers” have stopped growing just reaches that which corresponds to the liquid–liquid equilibrium binodal on which microscopic liquid–liquid phase separation takes place and not that which conforms to the fixing of the polymer.

The interpretation of our suggested structure formation mechanism is devoid of the above-indicated shortcomings and does not contradict the experimental data available, but its complete validation requires further investigation.

### 3.5. Morphology of Fibers Formed from PLGA/TG Mixtures via NIPS

Figure 8 presents SEM photographs of transverse cleavages of PLGA fibers formed by the methodology described above in Section 2.2.4 from PLGA/TG mixtures containing 10, 20, and 30 wt.% PLGA.

Fibers having finger-like pores in their structure were found to form only from the mixtures containing 10 wt.% PLGA using nonsolvents containing up to 50 wt.% TG. This fully agrees with the results of our optical experiments presented in Section 3.3. The further “softening” of the nonsolvent led to the transformation of the finger-like pores into pores of irregular shape.

Fibers obtained from the mixtures containing 20 wt.% PLGA featured a spongy structure with a small number of macropores from 100 to 300 μm across localized along the central axis of the fiber. As the TG content of the nonsolvent was increased, the mean size of the macropores decreased only slightly. The absence, in this case, of the finger-like pores described in Section 3.3 (Figure 6b) is explained by the smaller radius of the fiber, compared to that of the samples investigated by us in the optical experiment. In fibers obtained from the mixtures with 20 wt.% PLGA, such pores are only observed at a distance of 100 μm from the surface of the fiber.

Fibers obtained from the 30 wt.% PLGA solution are basically characterized by a uniform porous structure (Figure 8d). The exclusion is the structure of the fibers formed using the most “harsh” nonsolvent, i.e., water. In that case, the formation of a nonporous layer on the surface of the fiber slows down the diffusion of water inside it to such a degree that droplets of the polymer-poor phase at the center of the fiber have enough time to coalesce (until the polymer-rich phase turns glassy) and form a central channel nearly a third of the total thickness of the fiber in diameter (Figure 8e). As already noted, this is associated with the formation of the less permeable surface structure and the small number and size of through-pores therein.

**Figure 9:**
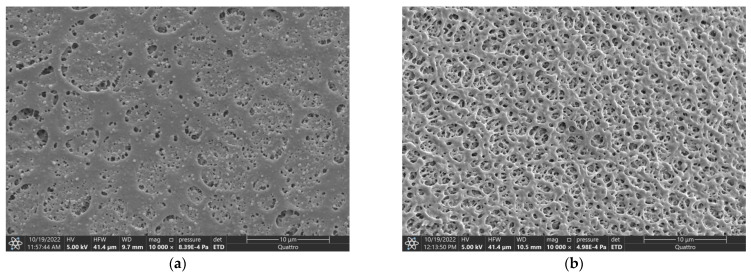
Surfaces of fibers formed from the 20 wt.% PLGA solution via immersion in (**a**) water and (**b**) water/TG (50:50) mixture.

The condition of the external surface of the fibers depends on the composition of the nonsolvent bath as well (Figure 9). Its porosity increases as the activity of the nonsolvent is decreased and as the initial polymer concentration is decreased. This agrees with the above description of the fiber formation mechanism associated with the development of the surface layer that hinders the subsequent mass-exchange processes. When the fibers are formed using a mixed water/TG nonsolvent instead of pure water, the number of pores in their surface layer substantially increases, and so does the mean size of the pores. In the case of fibers formed from the 20 wt.% PLGA solution, increasing the TG content of the composite precipitator from 0 to 50% led to a 2- to 3-fold increase in the mean pore size, from 1 μm (for pure water as the nonsolvent) to 2.5 μm (for the mixed nonsolvent). The results obtained bear out the supposition made above that the porosity of the surface layer depends on the extent of its enrichment in the polymer as a result of the extraction of the solvent from it into the nonsolvent. Indeed, since the extraction power as regards TG is higher in pure water than in the mixed water/TG nonsolvent, the surface porosity of fibers obtained via immersion in the mixed nonsolvent, as expected, was higher than that of their counterparts obtained by immersion in pure water.

## 4. Conclusions

During the course of our experimental investigations into the phase and structural transformations in the ternary PLGA/TG/water system, we obtained the following main results.

- A phase diagram of the above mixture at room temperature containing only a single boundary line—the liquid–liquid equilibrium binodal—is constructed for the first time.
- Using the DSC analysis technique, the PLGA/TG mixture composition, with which the polymer turns glassy at room temperature, is determined. The point corresponding to this composition (~95 wt.% PLGA + 5 wt.% TG) is plotted on the phase diagram.
- Using an improved methodology, the evolution of the structure of PLGA/TG mixtures containing 10, 20, and 30 wt.% PLGA immersed either in pure water or in a mixed water/TG (50:50) nonsolvent is studied for the first time. It is found that as the polymer content of the structure-forming mixture is increased, the structure formed transforms from a matrix with finger-like pores concentrated near its surface layer to matrices wherein such pores are located at some distance from this layer and, finally, to structures altogether devoid of such pores. Where use is made of the mixed nonsolvent instead of pure water, mixtures containing over 25 wt.% PLGA develop the only type of structure—a spongy (interconnected) structure—free from “fingers”.
- A new structure formation mechanism of the mixtures under study is suggested, according to which the architectonics of the structure formed as a consequence of the microscopic liquid–liquid phase separation of the PLGA/TG/water mixture depends on the barrier properties (morphology) of the surface layer that comes into existence at the interface between the contacting phases, with these properties being, in turn, dependent on the thermodynamic affinity of the solvent for the nonsolvent and the polymer content of the structure-forming mixture.

## Figures and Tables

**Table 1:** Hansen’s solubility parameters of the substances used in this work.

Substance	δ_d_, MPa^0.5^	δ_p_, MPa^0.5^	δ_H_, MPa^0.5^
PLGA	17.3	10.1	8.4
TG	17.8	10.0	12.7
Water	15.5	16.0	42.3

## Data Availability

Available upon request.
